# The complete mitochondrial genome of the black-breasted thrush *Turdus dissimilis* (passeriformes: Turdidae)

**DOI:** 10.1080/23802359.2023.2278826

**Published:** 2024-10-03

**Authors:** Xue Gou, Shize Li, Haijun Su

**Affiliations:** a Guizhou State-owned Longli Forest Farm, Longli, China; b College of Forestry, Guizhou University, Guiyang, China; c College of Life Sciences, Guizhou University, Guiyang, China; d Department of Food Science and Engineering, Moutai Institute, Renhuai, China; e Research Center for Biodiversity and Natural Conservation, Guizhou University, Guiyang, China

**Keywords:** Complete genome, gene arrangement, mitochondrial DNA, *Turdus dissimilis*

## Abstract

The Back-breasted Thrush (*Turdus dissimilis* Blyth 1847), a medium-sized *Turdus* bird in the Turdidae family, is widely distributed in montane areas from northeastern India and Myanmar to southern China. The mitochondrial *DNA* of *T. dissimilis* is packaged in a compact 16,761-basepair (bp) circular molecule with *A + T* content of 52.50%. It contains 37 typical mitochondrial genes, including 13 protein-coding genes, 2 *rRNAs* and 22 *tRNAs*, and 1 noncoding region. We reconstructed a phylogenetic tree based on the mitogenome sequences of 10 Turdidae species and one outgroup. Phylogenetic analysis indicated that *T. dissimilis* is a sister taxon to *T. unicolor.* The new mitogenome data would provide useful information for application in conservation.

## 1. Introduction

**Figure 1.**
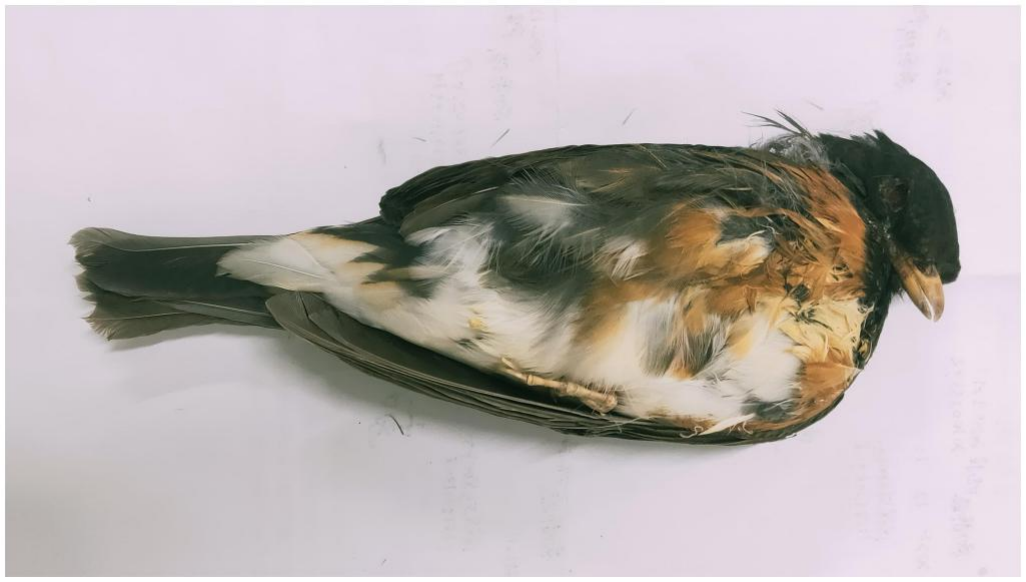
Image of *T. dissimilis* has a lower body orange brown, mouth and feet waxed yellow. The image taken by author Xue Gou in Guiyang China.

The Black-breasted Thrush (*Turdus dissimilis* Blyth 1847), a medium-sized *Turdus* bird in the Turdidae family, is widely distributed in East and South Asia. It breeds in montane areas from northeastern India and Myanmar to southern China and winters in China, Bangladesh, India, Laos, Myanmar, Thailand, and Vietnam (Pedrocchi 2015). Both males and females share a similar orange-brown underbody and a long, yellow beak (Figure 1). *T. dissimilis* mostly inhabits forests and shrublands, especially broad-leaved forests dominated by *Rhododendron* and mixed coniferous broad-leaved forests ranging from 1200 m to 2500 m a.s.l. (BirdLife International 2021).

*T. dissimilis* is listed as Least Concern (LC) (BirdLife International 2021). The population of this species is suspected to be in decline owing to habitat destruction and degradation (Jiang et al. 2016). Jiang (2018) has reported the complete mitogenome of some *Turdus* species, but no complete mitochondrial genome data of *T. dissimilis* is available in GenBank thus far. We sequenced the complete mitochondrial genome of *T. dissimilis* (GenBank number: MW307918) and examined its phylogenetic relationship with others.

## 2. Materials and methods

### 2.1. Sample collection and preservation

The complete mitochondrial genome of the *T. dissimilis* was sequenced using muscle tissue collected from Longdongbao Airport in Guiyang, China (106°40′19.60″E, 26°25′58.42″N), and a specimen was deposited in the Research Center for Biodiversity and Nature Conservation of Guizhou University, Guiyang, China (http://www.gzu.edu.cn; Xue Gou; 2136561724@qq.com) under the voucher number NO. GZUNZ20201129002.

### 2.2. DNA extraction and sequencing and phylogenetic analysis method

The extraction was performed using a DNA Rapid Extraction Kit (Beijing Aidlab Biotechnologies Co., Ltd) according to the kit manual. Before the DNA extraction, approximately 50 mg of muscular in *T. dissimilis* was cut with sterile scissors and again rinsed in sterile water. To amplify the mitochondrial genome of the black breasted thrush, the primers used in this study were designed based on the mt genome sequences of closely related species (Bo Li et al. 2016). The PCR amplification was mainly carried out through the LA-Taq polymerase reaction (Table 1). The sequence was submitted to the GenBank with the accession number MW307918.

The PCR conditions were as follows: initial denaturation at 94 °C for 2 min, then 35 cycles of denaturation at 94 °C for 30 s, annealing at 55 °C for 30 s, and extension at 72 °C for 1 min/kb, followed by the final extension at 72 °C for 10 min. The total volume for PCR and LA-PCR was 50 μl, of which Takara LATaq (5 U/μl) was 0.5 μl, 10 × LATaq Buffer II (Mg2+) was 5 μl, dNTP mixture (2.5 mM) was 8 μl, template was 60 ng, and the total volume was then made up with distilled water. The final concentration of the forward and reverse primers was 0.2 ∼ 1.0 μM, and that of MgCl2 was 2.0 mM.

According to the guidelines for Fast-DNA extraction kit preparation (Beijing Adlai Biotechnology Co., Ltd.), prepare a sample library for sequencing and sequence it on the ABI 3730 automatic sequencer (Sanger sequencing) (Zou et al. 2017; Zhang et al. 2018). After quality-proofing of the obtained fragments, the complete mt genome sequence was assembled manually using DNAstar v7.1 software (Zou et al. 2017; Zhang et al. 2018). Specifically, the original mitogenomic sequences were first imported into the MITOS web server to determine the approximate boundaries of the genes. Exact positions of protein-coding genes (PCGs) were found by searching for ORFs (employing genetic code 2, the vertebrate mitochondrion). All tRNAs were identified using ARWEN, DOGMA and MITOS (Wyman et al. 2004; Laslett and Canback 2008; Bernt et al. 2013). The precise boundaries of 12S rRNA and 16S rRNA were determined by comparison with homologs. PhyloSuite, an in-house GUI-based software, was used to parse and extract the information from genomes manually annotated in Word documents, as well as create GenBank submission files and organization tables for mitogenomes. Meanwhile, the mitogenome sequences of 10 species of Turdus and one outgroup were analyzed by phylogenetic maximum-likelihood tree using mega software.

## 3. Results and discussion

**Figure 2.**
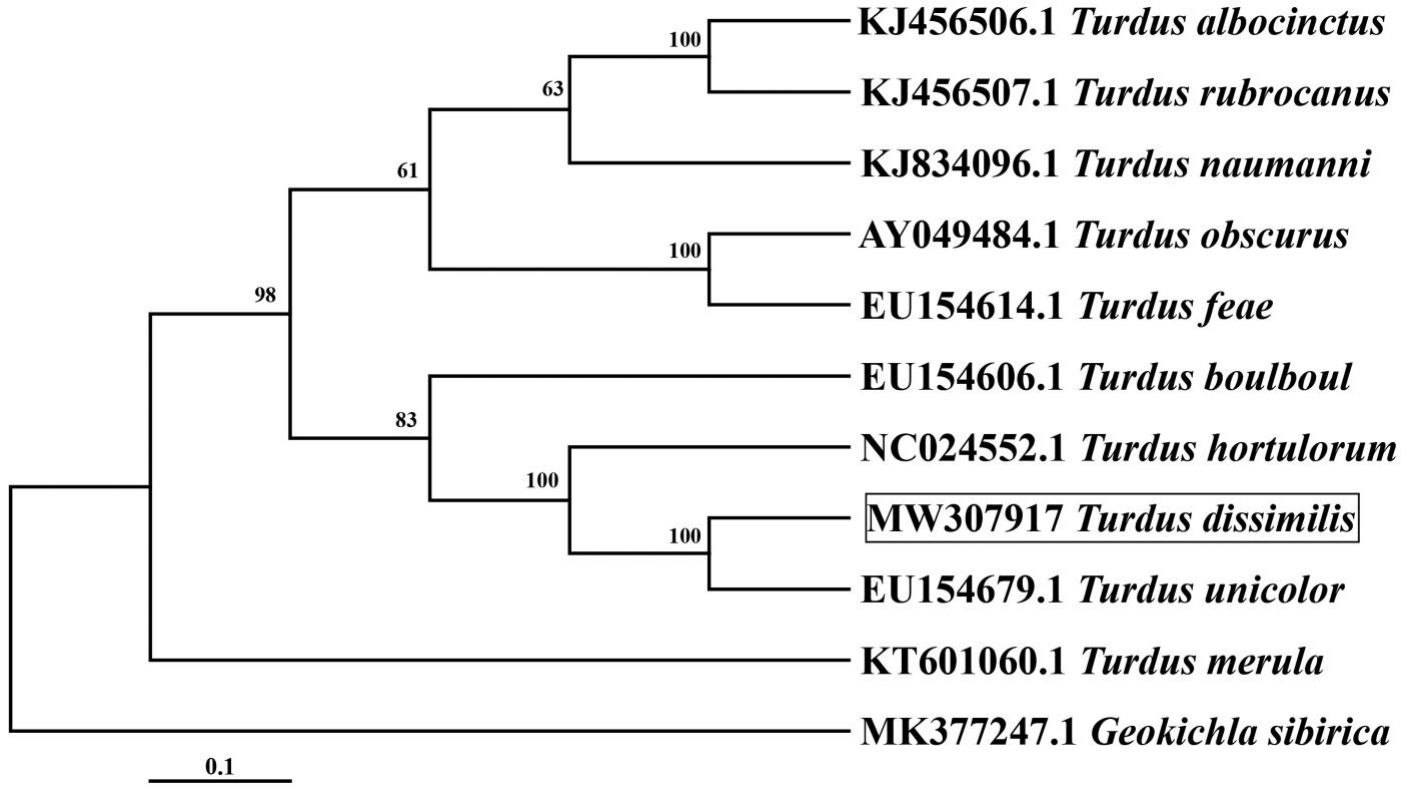
Phylogenic maximum-likelihood tree based on mitogenome sequences of 10 Turdidae species and one outgroup. The following sequences were used. KJ834096.1 *Turdus naumanni* (Bo Li et al. 2016), KJ456506.1 *Turdus albocinctus*, KJ456507.1 *Turdus rubrocanus*, AY049484.1 *Turdus obscurus*, EU154614.1 *Turdus feae*, EU154606.1 *Turdus boulboul*, NC024552.1 *Turdus hortulorum*, EU154679.1 *Turdus unicolor*, KT601060.1 *Turdus merula*, MK377247.1 *Geokichla sibirica*[Data sourced from NCBI database, Unpublished)].

The complete mtDNA sequence of *T. dissimilis* is 16,761 bp in length, with a base composition of A, 29.30%; C, 32.60%; G, 14.90%; and T, 23.20%. The A + T content accounted for 52.50% of the total, which is within the range of avian mitogenomes (51.6-55.7%; Haring et al. 2001; Table 2). It has a typical circular mitochondrial genome containing 13 protein-coding genes, 22 transfer RNAs, 2 ribosomal RNAs, and 1 noncoding A + T-rich region, which is usually found in birds (Boore 1999). The order and orientation are identical to the standard avian gene order (Gibb et al. 2007). Of the 13 protein-coding genes, 11 genes utilize the standard mitochondrial initiation codon ATG; however, COI and ND3 use GTG as the initiation codon. TAA is the most frequent stop codon, although COI ends with AGG, ND6 ends with TAG, ND1 and ND5 end with AGA, and COIII and ND4 end with the single nucleotide T. The 12S rRNA is 975 bp, and the 16S rRNA is 1598 bp in length, which are located between tRNA-Phe (gaa) and tRNA-Leu (taa) and separated by tRNA-Val (tac). All tRNAs possess the classic cloverleaf secondary structure, as observed in other bird mitogenomes (Bernt et al. 2013). Most of the mitochondrial genes are encoded on the heavy strand (H-strand), except for ND6 and eight tRNA genes, which are encoded on the light strand (L-strand). However, the tandem repeat analysis of the CR gene by Tandem Repeats Finder software showed that the tandem repeat number of the sample gene sequence was 0. This indicates that the genomic DNA in this region has no repeated gene sequences, or the length of the repeated sequence is less than the detection threshold. In addition, phylogenetic tree results indicated that T. dissimilis is the sister taxon of T. unicolor (Figure 2).

**Table 1.** Primers used for amplification of the mitochondrial genome of Turdus dissimilis.

Fragment No.	Gene or region	Primer name	Sequence (5’-3’)	Length (bp)
F1	12S-16S	HXF1	GCTAGCAAGACAGGTCAAGG	1681
		HXR1	CGTCGATATGGACTCTTGGA	
F2	16S-COX1	HXF2	GTTCACTTACACGTAAGCCAC	3323
		HXR2	CCACGTTGTAGATTTGGTCG	
F3	tRNA-Cys-tRNA-Ser	HXF3	CATGAACTTCACTACAGAGC	1664
		HXR3	CATATGGGGGTTCGACTCC	
F4	COX1-ATP6	HXF4	CATGCCTCGACGATACTCAG	1658
		HXR4	GTAGGCCTGTGAGGAGTGTTG	
F5	ATP6-tRNA-Arg	HXF5	CATTAACCTAACTACAAAGC	1770
		HXR5	CAACTGTCTTGGTTAGACTAAC	
F6	ND3-12S	HXF6	CCTAGTTGCCATCCTATTCC	8074
		HXR6	GTACACTTACCTTGTTACGAC	

**Table 2.** *Organization of the complete mitochondrial genome of Black-breasted Thrush* Turdus dissimilis.

Gene	Position Start-End	Size	Spacer (+) or Overlap (–)	Codon		Anti-codon	Strand
				Start	Stop		
*tRNA-Phe*	1–68	68				GAA	H
*12S rRNA*	69–1043	975					H
*tRNA-Val*	1044–1113	70				TAC	H
*16S rRNA*	1114–2711	1598					H
*tRNA-Leu*	2712–2786	75				TAA	H
*ND1*	2791–3768	978	4	ATG	AGA		H
*tRNA-Ile*	3775–3847	73	6			GAT	H
*tRNA-Gln*	3854–3924	71	6			TTG	L
*tRNA-Met*	3924–3992	69	−1			CAT	H
*ND2*	3993–5033	1041		GTG	TAA		H
*tRNA-Trp*	5033–5102	70	−1			TCA	H
*tRNA-Ala*	5104–5172	69	1			TGC	L
*tRNA-Asn*	5177–5251	75	4			GTT	L
*tRNA-Cys*	5252–5318	67				GCA	L
*tRNA-Tyr*	5318–5388	71	−1			GTA	L
*COI*	5390–6940	1551	1	GTG	AGG		H
*tRNA-Ser*	6932–7004	73	−9			TGA	L
*tRNA-Asp*	7008–7077	70	3			GTC	H
*COII*	7088–7771	684	10	ATG	TAA		H
*tRNA-Lys*	7773-–7840	68	1			TTT	H
*ATP8*	7842–8009	168	1	ATG	TAA		H
*ATP6*	8000–8683	684	−10	ATG	TAA		H
*COIII*	8687–9470	784	3	ATG	T		H
*tRNA-Gly*	9471–9539	69				TCC	H
*ND3*	9540–9890	351		ATG	TAA		H
*tRNA-Arg*	9892–9961	70	1			TCG	H
*ND4L*	9963–10,259	297	1	ATG	TAA		H
*ND4*	10,253–11,630	1378	−7	ATG	T		H
*tRNA-His*	11,631–11,700	70				GTG	H
*tRNA-Ser*	11,701–11,767	67				GCT	H
*tRNA-Leu*	11,767–11,837	71	−1			TAG	H
*ND5*	11,838–13,655	1818		ATG	AGA		H
*Cytb*	13,664–14,806	1143	8	ATG	TAA		H
*tRNA-Thr*	14,808-–14,877	70	1			TGT	H
*tRNA-Pro*	14,885–14,954	70	7			TGG	L
*ND6*	14,96515,483	519	10	ATG	TAG		L
*tRNA-Glu*	15,485-–15,556	72	1			TTC	L
*D-LOOP*	15,557–16,761	1205					

## 4. Conclusions

We report for the first time the complete mitochondrial genome of T. dissimilis and annotate and analyze its base composition, gene alignment sequence, PCGs, and secondary structure of tRNAs and rRNAs. The results showed that *T. dissimilis* is the sister taxon of *T. unicolor*. The results are beneficial to the conservation of T. dissimilis species and provide fundamental genetic data for the evolutionary research on Turdidae.

## Supplementary Material

Supplemental Material

Supplemental Material

## Data Availability

The genome sequence data that support the findings of this study are openly available in GenBank of NCBI at https://www.ncbi.nlm.nih.gov under the accession no. MW307918. The associated BioProject.PRJNA733139.
