# Supplementary figures and images for: The complete mitochondrial genome of the black-breasted thrush Turdus dissimilis (passeriformes: Turdidae)
Source: Mitochondrial DNA B Resour. 2024 Oct 3;9(10):1298–301. doi: 10.1080/23802359.2023.2278826 (PMC11457367; doi:10.1080/23802359.2023.2278826)

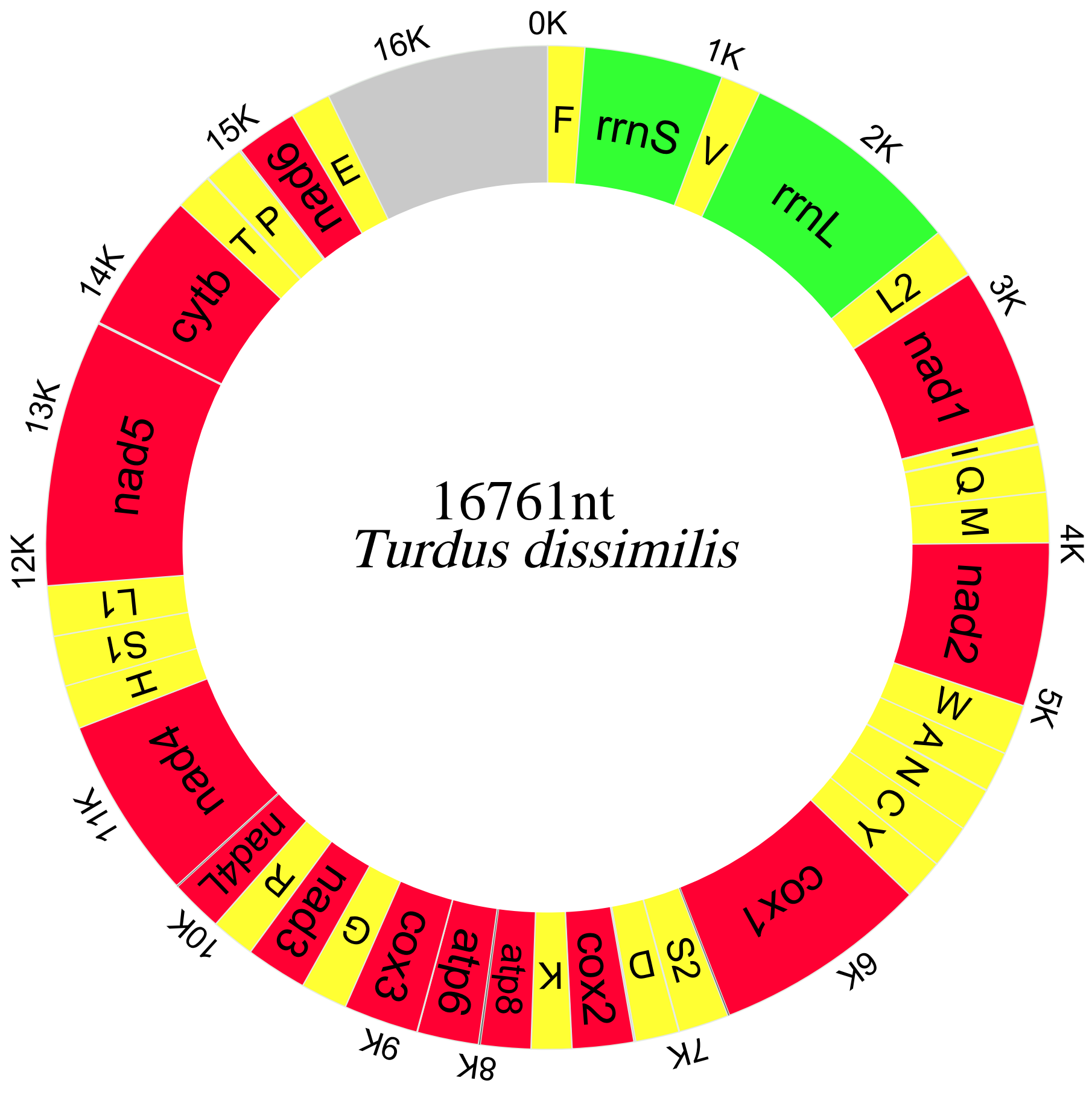

Supplement: Supplemental Material [file TMDN_A_2278826_SM5751.pdf]
