# Supplementary material for: The complete mitochondrial genome of the black-breasted thrush Turdus dissimilis (passeriformes: Turdidae)
Source: Mitochondrial DNA B Resour. 2024 Oct 3;9(10):1298–301. doi: 10.1080/23802359.2023.2278826 (PMC11457367; doi:10.1080/23802359.2023.2278826)
Supplement: Supplemental Material [file TMDN_A_2278826_SM5750.pdf]

贵州大学实验动物伦理审查申请表

Inspection Form for the Guizhou University Experimental Animal Ethics

编号（No）：EAE-GZU-2023-T105

一、申请项目基本信息 Project information

申请单位<br>Name of organization	茅台学院		
申请人<br>Applicant	苟雪	联系电话<br>Telephone	18788600928
项目/实验名称<br>Project/Experiment title	黑胸鸫的线粒体全基因组及其系统发育关系		
申请日期<br>Application date	2023 年 3 月 13 日	是否普通项目/实验<br>Is common project/experiment ?	是
拟实验时间<br>Experiment date	2023 年 3 月 18 至<br>2023 年 4 月1	是否重大项目<br>Is major project ?	否

二、使用动物 Animal information

动物品种/品系<br>Species or strains	黑胸鸫
动物等级(普通/濒危/极危)<br>Grades (ordinary, endanger, rare)	普通

三、实验要点 Experment information:

包括实验目的、实验方法、观测指标、实验结束后处死动物的方法等

Outline of experiments, including aim, methods, detection index, executing animal method, *et al*.

实验目的： 测量黑胸鸫的线粒体全基因组，探究其系统发育关系

实验方法： 实验过程中取黑胸鸫腿部肌肉，使用 DNA 快速提取试剂盒（北京艾德莱生物技术有限公司）按照试剂盒说明书进行提取，线粒体基因组用于设计聚合酶链式反应(PCR)的引物并用作基因注释的模板。在使用 Sanger 方法自动测序的 ABI 3730 上对有丝分裂基因组进行测序。

观测指标： 样本线粒体基因组成

处死动物的方法： 野外研究时发现意外死亡的动物样本（黑胸鸫），对样本进行杀菌消毒后采集需要的组织样本，剩余尸体保存于茅台学院生物学标本储藏室。

四、申请者承诺 Announcement of applicant

我将自觉遵守实验动物福利伦理原则，随时接受实验动物伦理委员会的监督与检查，如违反规定，自愿接受处罚。
I will abide by the rules of animal experimental ethics, accept the supervision and inspection of the animal experimental ethics committee, and accept the punishment in case of any infringement.)

申请人 (Applicant)：尚雪

2023年3月13日

五、审查依据 Inspection contents

1．该项目是否必须用实验动物进行实验，即能否用计算机模拟、细胞培养等非生命方法替代动物或用低等动物替代高等动物进行实验？(Does laboratory animal must be used in the project? Could other methods be used, such as computer simulation, cell culture or using the low-grade animal instead of the high-grade animal?)
2．表中所填申请人资格和所用动物的品种品系、质量等级、规格是否合适，能否通过改良设计方案来减少所用动物的数量？(Are the qualification of applicant, species or strain, grade and specifications of animals suitable? Could the quantity of animals be reduced by improving the study design?)
3. 能否通过改进实验方法、调整实验观测指标、改良处死动物的方法，来优化实验方案、善待动物？(Could the study design and animal treatment be refined by ameliorating experimental method, adjusting observational index, executing animal method?)

六、审查结果 Results of inspection

所在单位负责人意见 Opinion by the leader	同意 签名：[illegible] 2023年3月16日（公章）
贵州大学实验动物伦理分委员会意见 Opinion of the Guizhou University Subcommittee of Experimental Animal Ethics	同意 主任签名： 签章：贵州大学 Guizhou University 实验动物伦理分委员会 Subcommittee of Experimental Animal Ethics 2023年3月17日
备注: Remark	

说明：
1.申请表纸质版一式两份（双面打印）递交到贵州大学实验动物伦理分委员会办公室，编号由贵州大学实验动物伦理分委员会填写。
2.申请人需手写签名。
3.需随本表递交相关审查资料包括实验方案、课题标书（复印件）等。要求写明项目的意义、必要性、项目中有关实验动物的用途、饲养管理或实验处置方法、预期出现的对动物的伤害、处死动物的方法、项目进行中涉及动物福利的详细描述。
